# Treatment and Qualitative Research of Schizophrenia

**DOI:** 10.1192/j.eurpsy.2022.2025

**Published:** 2022-09-01

**Authors:** M. Ammon

**Affiliations:** German Academy for Psychoanalysis, Board, Head Of Training And Research, Berlin, Germany

**Keywords:** schizophrénia, Dynamic Psychiatry, Psychodynamic understanding

## Abstract

**Introduction:**

The author demonstrates the psychodynamic understanding of schizophrenia and describes the ensuing personality-structural psychotherapy. Schizophrenia from a psychodynamic understanding is a disease in the core of the identity with disturbances of the personality functions of identity, ego-demarcation, aggression, fear, narcissism, perception, cognitive abilities and the body- ego. It is the concern of the author to investigate how schizophrenically structured patients and their family members experience the group dynamic field in which the patients grew up and its relations to the illness. The following five topics: contact and experiences within the family of childhood, body care and physical contact, kindergarden and school life, puberty, and contacts outside the family have been investigated.

**Objectives:**

The aim is to show how the family settings and backgrounds are conducive to developing schizophrenia

**Methods:**

The author chosed for her investigation the method of biographical interviews, introduced by Witzel (1985). This method of interviewing is problem centered, object and process oriented. The analysis of the exhaustive tape-recorded interviews was made by using the method of qualitative analysis based on the grounded theory.

**Results:**

Schizophrenia from psychodynamic understanding is a disease in the core of the identity with disturbances of the personality functions of identity, ego-demarcation, aggression, fear, narcissism, perception, cognitive abilities and the body- ego.

**Conclusions:**

It is the concern of the author to investigate how schizophrenically structured patients and their family members experience the group dynamic social energetic field in which the patients grew up and its relations to the illness

**Disclosure:**

No significant relationships.

